# Analysis of Density Changes of Selected Brain Receptors After a 14-Day Supply of Chromium(III) and Evaluation of Chromium(III) Affinity to Selected Receptors and Transporters

**DOI:** 10.1007/s12011-019-01924-y

**Published:** 2019-11-16

**Authors:** Anna Piotrowska, Agata Siwek, Małgorzata Wolak, Gabriel Nowak

**Affiliations:** 1 Department of Biochemistry and Basics of Cosmetology, University of Physical Education, al. Jana Pawła II 78, 31-571 Kraków, Poland; 2 Department of Pharmacobiology, Jagiellonian University Medical College, Kraków, Poland; 3 Department of Neurobiology, Laboratory of Trace Elements Neurobiology, Institute of Pharmacology PAS, Kraków, Poland

**Keywords:** Chromium, Adaptative changes, Pharmacological effect, Mood disorders, Major depression

## Abstract

Chromium(III) is one of the most controversial biometals. Although, it is no longer on the list of minerals necessary for the proper functioning of the human body, and its pharmacological effect is still under discussion. One of the purposes of Cr(III) administration is to use it in patients with mood disorders and it is strictly related to its pharmacological, not dietary effect. This is because its high doses are necessary to obtain the results and additionally, no deficiencies in human population have been noted. In this study, the affinity of chromium(III) to selected receptors and transporters in the rat brain was evaluated, and the effect of the 14-day administration of this metal was assessed on the density of selected receptors. All analyses were performed in vitro using radioligand binding assays, and the results indicated lack of affinity to β_1_ and α_1_ receptors and serotonin transporter (SERT), furthermore very weak affinity to the 5-HT_1A_ receptor (30% inhibition at 10^−4^ and 10^−5^ M). Analysis of the α_1_ and β_1_ adrenergic receptor density indicated lack of any adaptive effects after 14 days of Cr(III) administration through intraperitoneal injections (doses 6 and 12 mg/kg). The antidepressant activity of chromium(III) indicated in clinical trials concerned patients with atypical, seasonal, or dystonic symptoms. This effect, as it seems based on the presented results, does not depend on direct affinity to serotonin receptors and transporter nor is the result of adaptive changes in the adrenoreceptor system.

## Introduction

Currently, chromium(III) is probably the most controversial biometal, and concepts about its role are still changing. In 2014, it was removed from the list of micronutrients [1]. Since then, the evidence that Cr(III) is not a micronutrient have multiplied [2, 3].

The basic biological properties of Cr(III) relate to its activities in the metabolism of carbohydrates [4], which, apart from the use in supplementation of patients in various states of impaired glucose utilization, allows using Cr(III) supplementation in patients with PCOS [5]. Other directions of usage were also indicated in available literature. The possibility of influencing the dynamics of the development of cognitive disorders [6], post-stroke protective action [7], and the possibility of using or strengthening the pharmacotherapy of depression, especially atypical, was investigated [8–12]. The first postulates indicating the possibility of antidepressant effect of Cr(III) were established in 1994 [8, 9]. As it was shown in studies using behavioral tests (a modified swimming test in rats and mice) [13–15] and animal models of depression [16], this is due in varying degrees to effects on noradrenergic, dopaminergic, serotonin, and GABAergic receptors.

The results of the available research are not consistent. Piotrowska et al. [15], in contrast to the results of Khanam and Pallai [13], indicate the participation of the noradrenergic pathway in the antidepressant action of Cr(III). Studies in the mouse model have shown that this activity is partially dependent on the 5-HT_1A_ and 5-HT_2A_ receptors [14] and the dopaminergic system [15]. On the other hand, the noradrenergic mechanism was indicated by antagonism of Cr(III) action caused by combined administration with adrenergic receptor antagonists (propranolol, prazosin, yohimbine) and augmentation of reboxetine action [15]. It was also conveyed in studies by Franklin and Odontiadis [17] and Attenburrow et al. [18], where higher serotonin levels and downregulation 5-HT_2A_ serotonin receptors have been shown. The participation of the glutamatergic system (AMPA and NMDA receptors) in the FST test in mice was demonstrated by Piotrowska [14]. Khanam and Pillai [13] suggested the involvement of the K^+^ channel. Prof. Khanam’s group also indicates that chromium may have an anxiolytic effect and is the consequence of the activation of the serotonin pathway [19]. In addition, a decrease in the concentration of corticosterone in the blood plasma of animals subjected to chronic unpredictable mild stress was observed [16].

Clinical observations of the antidepressant action of chromium(III) salts concern primarily atypical depression, premenstrual syndrome (PMS) and premenstrual dysphoric disorder (PDD), seasonal affective disorders (SAD), and circadian mood swings [10, 11]. This profile of activities is interesting, and range of impact is slightly different for most current antidepressants.

The influence of chronic Cr(III) administration on the density of brain receptors has not been indicated so far. Chromium(III) affinity to these structures has not been studied. Therefore, the aim of the study was to determine the affinity of Cr(III) to selected receptors and transporters in rat brain and to evaluate the effect of chronic administration of this salt on the density of β_1_-adrenergic and α_1_-adrenergic receptors.

## Materials and Methods

All procedures were approved by the Local Ethical Committee of the Jagiellonian University Medical College, Kraków (112/2009). The experiments were carried out on adult male Wistar rats (180–250 g). The animals were housed under conditions with constant temperature (20–22 °C), a controlled 12:12 light-dark cycle and free access to standard pellet diet and tap water. Each experimental group consisted of 10 animals.

Imipramine (15 mg/kg, Sigma-Aldrich, Germany) and chromium(III) trichloride (CrCl_3_ × 6 H_2_O; 6 mg/kg and 12 mg/kg, Sigma-Aldrich, Germany) were administered *ip* everyday between 9.00 and 11.00 a.m. for 14 days. All compounds were dissolved in 0.9% saline. Control group of animals received an *ip* injection of saline (vehicle). The volume of vehicles or drug solutions for *ip* administrations was 2 ml/kg. Doses of chromium(III) were expressed per elemental Cr and selected on the basis of Franklin and Odontiadis research and were recalculated due to the use of other salts [17]. In connection with intraperitoneal administration, the dose estimation did not consider differences in oral absorption of chromium(III) chloride and chromium(III) picolinate.

Twenty-four hours after the last injection, rats were killed by decapitation. The cortex and hippocampus separately were rapidly removed from the brain, placed on dry ice, and then transferred into a − 80 °C freezer until use.

### Radioligand Binding Assay

#### β_1_-Adrenergic Receptors: Saturation and Inhibition Experiments

Rat cerebral cortex was homogenized in 20 volumes of ice-cold Tris-HCl buffer, pH = 7.6 in 25 °C using Ultra Turrax T25B homogenizer (IKA) and centrifuged at 20,000*g* for 20 min. After decantation of the supernatant, the pellet was resuspended in 20 volumes of buffer and centrifuged again in the same conditions.

Samples consisted of 240 μl of the tissue suspension (10 mg of wet weight), 30 μl of [^3^H]CGP-12177 (4-[3-[(1,1-dimethylethyl)amino]2-hydroxypropoxy]-1,3-dihydro-2H-benzimidazol-2-one hydrochloride, β_1_/β_2_ antagonist) in concentrations ranged from 0.03 to 3 nM were used to estimate the density of β_1_-adrenergic receptors. The inhibition experiments were performed in the same conditions but the concentration of radioligand was 0.2 nM.

To evaluate non-specific and total binding, 1 μM propranolol and buffer were used respectively. Samples in duplicate were incubated in 37 °C for 60 min. The incubation was finished by rapid filtration through Whatman GF/C filters and washed using a 96-well FilterMate Harvester (Perkin Elmer, USA). Radioactivity was measured using MicroBeta TriLux—liquid scintillation counter (Perkin Elmer). The protein concentration was estimated using Bradford reagent.

#### α_1_-Adrenergic Receptors: Saturation and Inhibition Experiments

Rat cerebral cortex was prepared according to previous procedure. Incubation mixture consisted of 240 μl of the tissue suspension (10 mg of wet weight), 30 μl of 0.186–3.5 nM [^3^H]prazosine, and 30 μl of buffer or 10 μM phentolamine to estimate total and non-specific binding respectively. For inhibition experiment, the concentration of radioligand was 0.2 nM.

Samples in duplicate were incubated for 30 min in 25 °C, filtered rapidly through Whatman GF/B filters, and washed with ice-cold buffer. Radioactivity was measured using MicroBeta scintillation counter (Perkin Elmer).

#### 5-HT1A Serotonergic Receptors: Inhibition Experiments

Rat hippocampi were homogenized in 20 volumes of ice-cold Tris-HCl buffer (pH = 7.7 in 25 °C). The homogenate was centrifuged in 10,000*g* for 10 min. Pellet was resuspended, incubated in 37 °C for 10 min, and centrifuged again. Reaction mixture consisted of 30 μl of 0.01 mM or 0.1 mM CrCl_3_, 30 μl of 1 nM [^3^H]8-OH-DPAT ((±)-8-hydroxy-2-dipropylaminotetralin hydrobromide, selective 5-HT1A agonist), and 240 μl of tissue suspension (5 mg wet weight/ml) was incubated in 37 °C for 20 min. Incubation was finished by rapid filtration through Whatman GF/B filters and washing with Tris-HCl buffer. All assays were performed in duplicate. A total of 10 μM serotonin was used to estimate non-specific binding.

#### Testing the Affinity of Chromium(III) to the Serotonin Transporter (5HT-T)

Rat cortex was homogenized in 20 volumes of ice-cold Tris-HCl buffer pH = 7.7 containing 120 mM NaCl and 5 mM KCl and centrifuged in 20,000*g* for 20 min. Pellet was resuspended and centrifuged twice in the same conditions. Reaction mixture consisted of 30 μl of 0.1 or 1 mM CrCl_3_ and 30 μl of 1 nM [^3^H]citalopram, and 240 μl of tissue suspension was incubated in RT for 1 h. Rapid filtration and washing with buffer were performed through Whatman GF/B filters. All assays were performed in duplicate.

### Statistical Analysis

The results were analyzed in GraphPad Prism 4, submitted as means ± standard deviations. The type of distribution was tested using the Shapiro-Wilk test; to detect outliers, the Grubbs test was used. Differences between groups, while meeting the requirements for the chosen test, were examined by a one-way analysis of variance using the Dunnet test as the post hoc test. The significance level *α* = 0.05 was assumed.

## Results

Analysis of chromium(III) affinity to selected receptors and transporters as a pilot study was performed in two points using high concentrations of the examined biometal (10^−3^–10^−5^). At selected concentrations, Cr(III) did not bind (α_1_, β_1_) or was bound extremely weakly (5-SERT, 5-HT_1A_). The results are shown in Table 1.

**Table 1:** Percentage of inhibition of a labeled ligand from its specific ligand-receptor complex

Receptor	Radioligand	Cr concentration [M]	% of inhibition
SERT	[^3^H]citalopram	10^−3^	3%
		10^−4^	0%
5-HT_1A_	[^3^H]8-OH-DPAT	10^−4^	30%
		10^−5^	30%
β_1_	[^3^H]CGP-12177	10^−3^	0%
		10^−4^	0%
α_1_	[^3^H]prazosin	10^−3^	0%
		10^−4^	0%

**Fig. 1:**
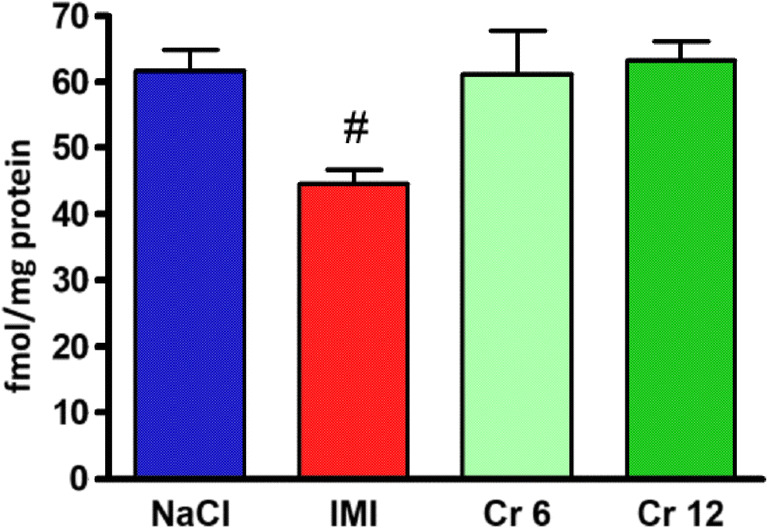
Beta-adrenergic receptor density in frontal cortex of rats after chronic administration of chromium (Cr 6 in dose 6 mg/kg or Cr 12 in dose 12 mg/kg) or imipramine (IMI) expressed as the femtomole per milligram of protein. The result is given as mean ± SEM (*n* = 5). #*p* < 0.05 vs. control group (NaCl). Post hoc test: Dunnet

**Fig. 2:**
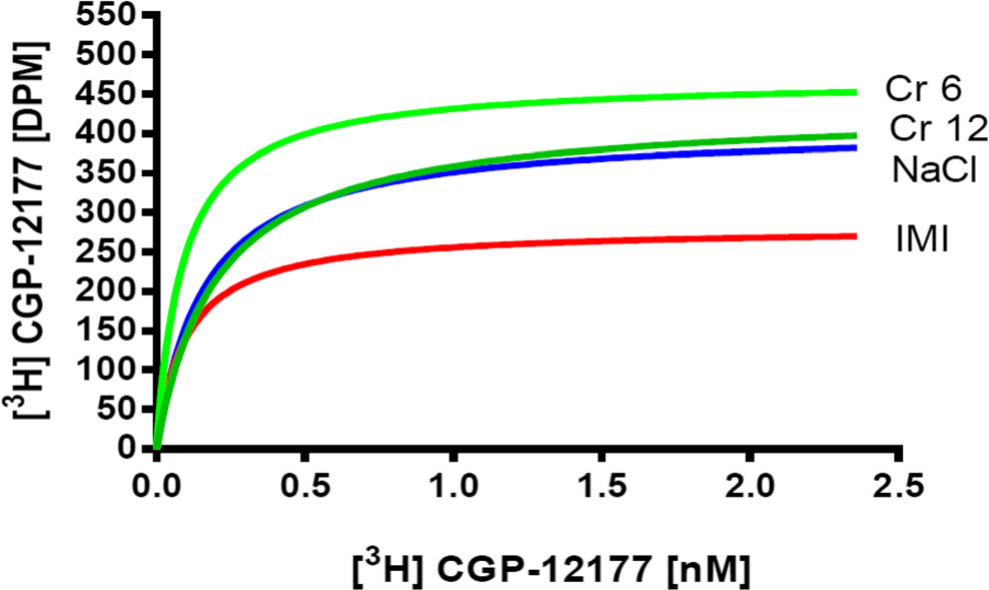
A representative result of saturation analysis for β-receptors. IMI imipramine, NaCl saline, Cr 6 chromium chloride in dose 6 mg/kg, Cr 12 chromium chloride in dose 12 mg/kg

The results of the saturation tests were subjected to one-way analysis of variance, and the presence of statistically significant differences in β-receptor density in the cortices of the rats subjected to 14 days of treatment (*F*_(3,16)_ = 4723; *p* = 0.0152). The tissues of imipramine-treated animals showed a reduced density of β-receptors (*p* < 0.05). Tissues of animals receiving chromium(III) salts in both doses (6 and 12 mg/kg) did not differ significantly from control tissues (Figs. 1 and 2).

**Fig. 3:**
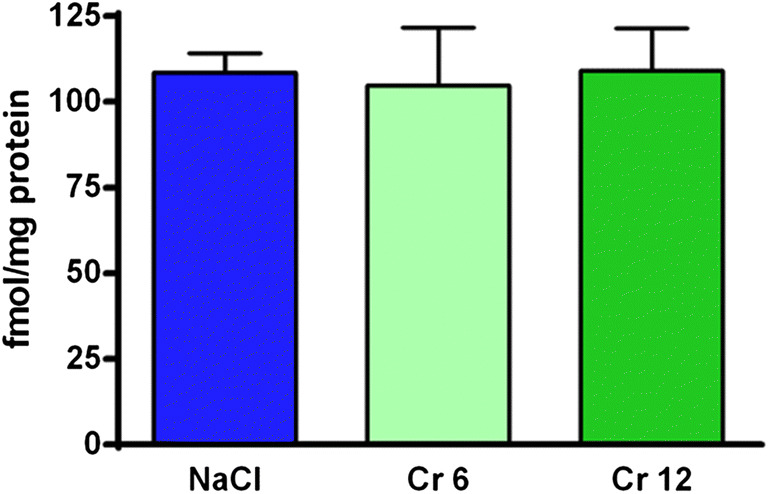
Density of α_1_-adrenergic receptors in the cortex of rats after chronic administration of chromium (Cr 6 in dose 6 mg/kg or Cr 12 in dose 12 mg/kg) or saline (NaCl). The result is given as mean ± SEM (*n* = 5)

**Fig. 4:**
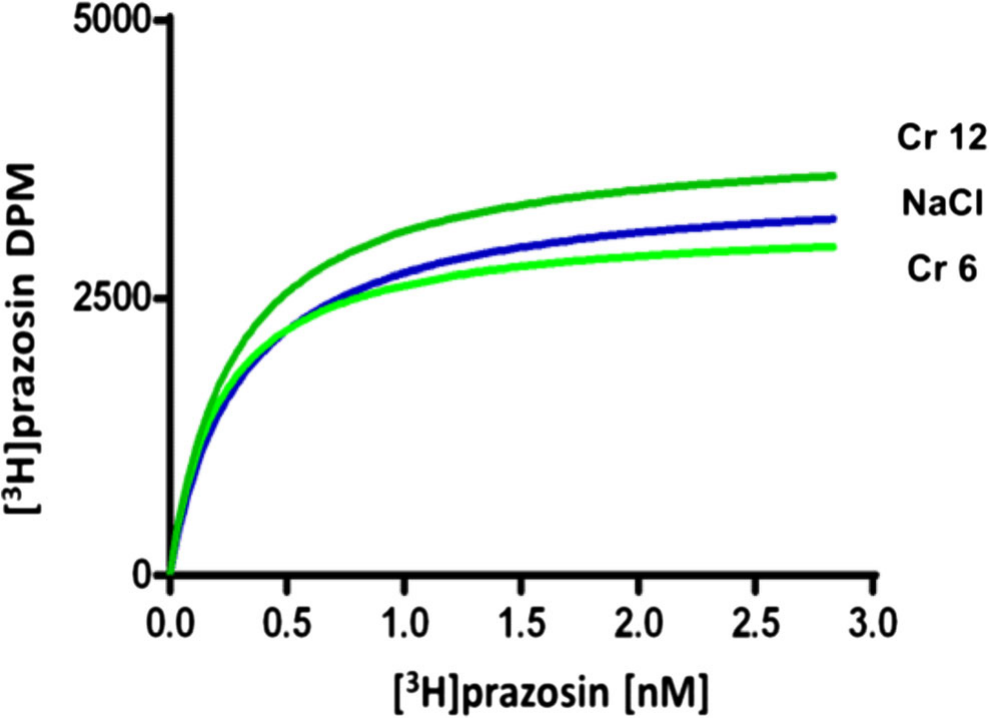
A representative of the saturation analysis for α_1_-receptor. NaCl saline, Cr 6 chromium chloride in dose 6 mg/kg, Cr 12 chromium chloride in dose 12 mg/kg

Alpha_1_ receptor density analysis after 14 days of therapy is shown in Fig. 3. The examined cortex did not show statistically significant differences (*F*_(3.16)_ = 0.03259 *p* = 0.9918). Figure 4 is an example of the result.

## Discussion

Understanding the mechanism of action of the antidepressant drug may indicate the research direction (new, or already tested and partially confirmed) for sources of disease, which, in the case of depression, is still the current topic. The association of a clinical picture that for depression may be inhomogeneous with the neurobiological mechanism of action of an antidepressant may allow the selection of a group of patients who are more likely to react properly to the prescribed drug. Clinical trials with the use of Cr(III) supplementation were used in patients with seasonal affective disorder, associated with the menstrual cycle, atypical depression, and dysthymia [10–12, 20, 21]. Patients responded favorably to monotherapy [12] and as augmentation of therapy [20].

In this study, the affinity of chromium(III) to selected receptors and transporters was examined, which is used as a screening method for assessing the suitability of new psychiatric treatments and the effect of chronic administration on the density of selected receptors in the cortex and hippocampus, and can be considered as an indicator of possible antidepressant effect. In our analyses, we have indicated that Cr(III) has no affinity to α_1_ and β_1_ adrenergic receptors.

Activity to α_1_ and β_1_ adrenoreceptors is not an intentional mechanism of action of antidepressants. It can also cause side effects. Lack of direct affinity to the α_1_ receptors may indicate that Cr(III) will not cause excess sedation, which has never been indicated as an adverse effect when using supplements with this metal [10–12, 20, 21]. On the other hand, blocking the α_1_ adrenoceptor may contribute to development of adaptative changes such as up-regulation, and thus changes desired from a therapeutic point of view.

The role of noradrenaline in the pathophysiology of depression has been repeatedly confirmed [for review: 22]. The individual receptors of the noradrenergic system differ in their function in the aspect of the mechanism of action of antidepressants. Blockade of α_1_ receptors mimics the symptoms of a depressive state (which, like chronic stress, may be associated with desensitization of these receptors) [23]. Some antidepressants show high affinity to the α_1_ receptors (amitriptyline, mianserin) and α_2_ (doxepin, imipramine, mirtazapine, or nortriptyline), which is their characteristic feature after single administration. Completely different effects occur after chronic administration. Such therapy, like repeated electroconvulsive treatments, increases the density of α_1_ receptors in the frontal cortex and hippocampus [22, 24], while the number of α_2_ receptors decreases [22, 25].

Adaptive changes of α_1_ and β_1_ receptors have been repeatedly used as one of the markers of antidepressant activity of newly discovered drugs. The first detected adaptive change was β-downregulation [26]. This effect is also caused by electroconvulsive shock (ECS) and most antidepressants (TCAs, MAOIs) except for SSRI, for which results are mixed [27]. In the noradrenergic system, up-regulation of the α_1_ receptors is also observed. This phenomenon has been confirmed by behavioral and electrophysiological studies and is caused by a variety of antidepressants [28–31] and ECS [24]. Currently used drugs (e.g., SSRI) do not give adaptive changes to adrenergic receptors.

Analysis of the density of selected receptors in the frontal cortex of the rats, after chromium(III) chloride administered at doses of 6 and 12 mg/kg for 2 weeks, gave negative results. In the examined tissues, there was no reduction in the density of β-receptors nor was there any increase in the density of α_1_ receptors. It is known that not all drugs with clinically proven antidepressant activity change the density of adrenergic cortical receptors (e.g., SSRIs do not induce β-downregulation).

Binding to serotonin receptors and serotonin transporter gave rise to several clinically useful groups of antidepressants. Unlike the earlier TLPD, SSRIs acted to raise levels of serotonin without increasing norepinephrine levels. The most drug-effective in this group is escitalopram. Its precursor, citalopram, has the properties of the most selective inhibitor of the serotonin transporter and the use of only one of the enantiomers further improved the selectivity [32].

In animal models, it was found that inhibition of SERT as well as the 5-HT_1B_ receptor increases the serotonin concentration in the prefrontal cortex, while agonists of this receptor increase the rate of firing of serotonergic neurons in the raphe nucleus [33]. This research direction allows new drugs to be introduced into the clinic, e.g., vortioxetine [34]. In presented study on the affinity of Cr(III) to selected receptors and transporters, no affinity to SERT, α_1_, and β_1_ was found; very poor activity was determined in relation to the 5-HT_1A_ receptor.

5-HT_1A_ are metabotropic receptors coupled to Gi/o proteins. Their activation lowers cyclic adenosine monophosphate (cAMP) concentration that leads to the inhibition of neuronal activity. 5-HT releasing is regulated by 5-HT_1A_ autoreceptors by the negative feedback mechanism in nuclei neurons. 5-HT_1A_ autoreceptors are associated with the pathophysiology of anxiety behavior. SSRI treatment increases 5-HT concentration in synaptic cleft leading to chronic activation of autoreceptors and in consequence to their desensitization. After several weeks, it effects in further alterations in the firing rates of the serotonergic neurons [35]. Mice with low 5-HT_1A_ autoreceptor expression are more sensitive to stress and respond to SSRI treatment faster than mice with higher expression of these receptors [36]. Authors show that raphe 5-HT_1A_ autoreceptors have negative influence on 5-HT release. In our research, the affinity of Cr(III) to the 5-HT_1A_ receptors has been indicated, but in the hippocampus, where they act as postsynaptic receptors.

Postsynaptic 5-HT_1A_ are heteroreceptors located in amygdala, hippocampus, septum, thalamus, and hypothalamus and response to 5-HT released in these regions [37–39]. There are several lines of evidence that 5-HT_1A_ heteroreceptors are involved in the behavioral response to antidepressant treatment. SSRIs are not active in NSF (novelty-suppressed feeding) test in 5-HT_1A_ knockout mice. In wild-type mice, the behavioral effect of antidepressants in NSF test occurs after chronic treatment with 5-HT_1A_ agonist—8-OH-DPAT. Administration of 8-OH-DPAT exerts antidepressant-like influence on adult hippocampal neurogenesis [40].

The studies of Samuels et al. [38] showed that deletion of 5-HT_1A_ receptors from mature dentate gyrus granule cells abolished the behavioral effect of the fluoxetine in mice. The neurogenesis and expression of hippocampal neurotrophic factor (BDNF and VEGF) as a result of SSRI administration were attenuated in mice lacking 5HT_1A_ receptors. Certain metals appear to affect the expression of BDNF (zinc, magnesium) [41, 42]. Such actions for chromium(III) have not been confirmed yet.

The use of Cr(III) in the therapy of psychiatric disorders concerns disorders with a complex manifestation. For today, the mechanism of positive effect of supplementation with this metal remains unclear. However, one should exclude direct action on the receptors of the serotonergic and noradrenergic systems and generation of adaptive changes in the latter system.

## Conclusions

Chromium(III) after 14 days of administration did not cause adaptive changes within the α_1_ and β_1_ receptors in rat’s brain. The ions of this metal have no affinity to the α_1_ and β_1_ receptors. The tested biometal has no affinity to the serotonin transporter (SERT) and the affinity of chromium(III) to the 5-HT_1A_ receptors is very low.

### Limitations

In this experiment, intraperitoneal injection was used due to the fact that chromium(III) chloride is absorbed very poorly form the gut. Data on the penetration of this metal through the blood–brain barrier is limited. The main limitation of this work is lack of brain Cr(III) level estimations.
